# Investigation of the effects of graded models on the biomechanical behavior of a bone-dental implant system under osteoporotic conditions

**DOI:** 10.12669/pjms.292.2963

**Published:** 2013-04

**Authors:** Ying Li, Zhong Shuang Liu, Xiao Ming Bai, Bin Zhang

**Affiliations:** 1 Ying Li, DDS, Institute of Hard Tissue Development and Regeneration, the Second Affiliated Hospital of Harbin Medical University, Harbin 150001, China.; 2 Zhong Shuang Liu, DDS, Institute of Hard Tissue Development and Regeneration, the Second Affiliated Hospital of Harbin Medical University, Harbin 150001, China.; 3 Xiao Ming Bai, PhD, Department of Astronautic Science and Mechanics, Harbin Institute of Technology, Harbin 150001, China.; 4 Bin Zhang, PhD. DDS, Institute of Hard Tissue Development and Regeneration, the Second Affiliated Hospital of Harbin Medical University, Harbin 150001, China.

**Keywords:** Cortical bone, Graded model, Biomechanical behavior, Finite element analysis

## Abstract

***Objective: ***To investigate the effects of graded models on the biomechanical behavior of a bone-implant system under osteoporotic conditions.

***Methodology*** *: *A finite element model (FEM) of the jawbone segments with a titanium implant is used. Two types of models (a graded model and a non-graded model) are established. The graded model is established based on the graded variation of the elastic modulus of the cortical bone and the non-graded model is defined by homogeneous cortical bone. The vertical and oblique loads are adopted. The max von Mises stresses and the max displacements of the cortical bone are evaluated.

***Results: ***Comparing the two types of models, the difference in the maximum von Mises stresses of the cortical bone is more than 20%. The values of the maximum displacements in the graded models are considerably less than in the non-graded models.

***Conclusions: ***These results indicate the significance of taking into account the actual graded properties of the cortical bone so that the biomechanical behavior of the bone-implant system can be analyzed accurately.

## INTRODUCTION

Dental implants have been extensively used in oral rehabilitation as replacements of lost natural teeth to restore human mastication functions. The quality of the jawbone was thought to be very important to the success of dental implants.^1^ It is well known that osteoporosis is a common bone disease that produced low-quality bone, and osteoporosis of the jawbone often results in the failure of dental prosthetics. Therefore, it is important to build reasonable mechanical models of the osteoporotic jawbone so that the biomechanical behavior of the bone-implant system can be simulated effectively and accurately.

The cortical microstructure contains Haversian canals, lacunae and vascular canals, which are defined as pores or cavities of the cortical bone. It has been shown by Parnell et al.^2^ that an obviously graded variation in the porosity in the cross section of the cortical bone exists. Moreover, this variation in porosity is usually heterogeneous at all ages. The results of a recent study showed that the porosity of the inner portion of the cortical bone increases with age.^3^ This finding has been confirmed in the animal trial of Dvorak at al.^4^ They found that cortical porosity was higher in osteoporotic sheep than in adult controls and that the changes became more pronounced when the histomorphometry was restricted to the inner millimeter of the cortical bone.

Increased porosity may significantly affect the material density and mechanical properties of bone. Schaffler and Burr^5^ found that the cortical bone stiffness was decided by its porosity and that significant increases in the porosity led to the decline of the elastic modulus. Bell et al.^6^ noted that there was an obvious negative impact on the cortex's ability to withstand stress with increased porosity.

Finite element analysis (FEA) is an acceptable research tool for the prediction of the stresses in an implant and its surrounding bone.^7^ However, the definition of the mechanical properties of the bone in the finite element models may influence the accuracy of the FEA results significantly.^8^ In most reported studies, the jawbone is usually assumed to be a system composed of a trabecular bone core surrounded by a cortical layer with a constant thickness.^9^ Both types of bones are described as a homogeneous material with constant moduli. Moreover, cortical bone is frequently modeled by assuming a constant elastic modulus across the thickness of the cortical bone.^10^ However, cortical bone has been found to be a naturally graded material with varied mechanical properties along the thickness direction in a number of studies in the literature.^11^ In particular, the investigations of Guo et al^12^ have shown that graded material properties may cause significantly different effects on material fracture behavior. Thus, assuming a constant material property cannot truly reflect upon the mechanical properties of bone tissue.

The aim of this study was to compare the biomechanical behavior of a bone-implant system under osteoporotic conditions using graded models and non-graded models to simulate the cortical bone, so that the effects of the graded models on the biomechanical behavior of the bone-implant system with an osteoporotic jawbone can be identified.

## METHODOLOGY

**Fig.1:**
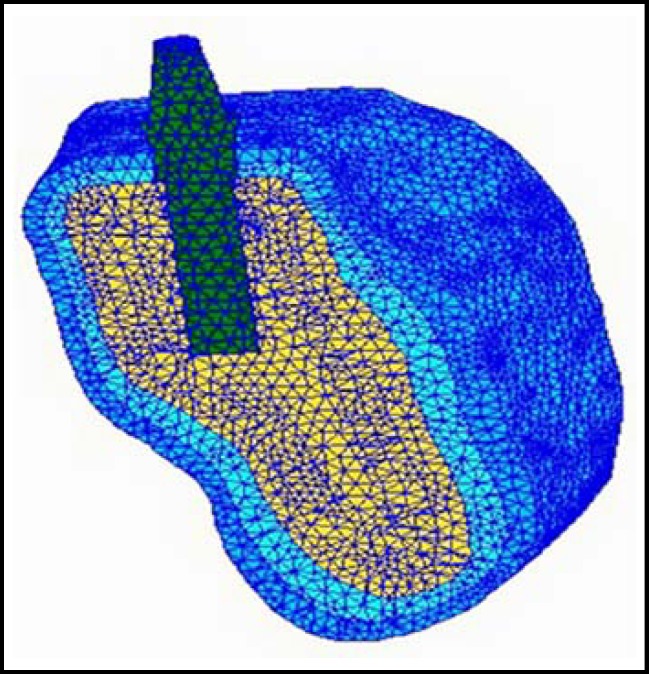
Cross-sectional view of the plane of the meshed model. The deep blue part represents outer layer of the cortical bone; the light blue part represents inner layer of the cortical bone; the yellow part represents trabecular bone

***Finite Element Model*** *: *The image is obtained from computed tomography (CT) scans, and the resulting stack of slices is imported into the software AMIRAE. The voxel data are segmented into volume sets representing the jawbone. The three-dimensional geometrical model of the implant-bone system is created by using the SOLIDWORKS software. The geometries of the implant presented in this article are extracted from Yang and Xiang^10^ and Wang et al.^13^ According to the combined solid model, a finite element model is established by using the MSC/PATRAN & NASTRAN software. A FEM of the posterior jawbone segments with a cylindrical threaded implant is shown in Fig.1.

***Elements, Nodes and Convergence Test*** *: *The models are meshed with 10-node tetrahedron elements. A fine mesh is generated around the implant. As shown in Fig.1, the whole system is meshed with 548,266 elements and 110,997 nodes. The convergence of the finite element models has been tested to guarantee the accuracy of the numerical results.

***Boundary Conditions and Loads*** *: *The models are constrained in all directions at the nodes on the mesial and distal bone surfaces. The estimated range of the force in a complete dentition is approximately 20–200 N.^13^ Moreover, Graf and Aeberhard^14^ found the ratio of vertical, oblique, and horizontal forces during chewing to be 5:2.5:1. Thus, vertical and oblique loads are adopted in this study. A 100 N vertical force is applied as a uniform pressure on the top surface of the abutment. An oblique load is applied with 100 N vertical and 30 N buccolingual components.

***Definition of Graded Models and Non-graded Models*** *: *Both types of models (a graded model and a non-graded model) with exactly the same geometry and mesh but different modeling methods for the material properties are established. The cortical and trabecular bones are modeled as transversely isotropic and linearly elastic materials in both types of models. In the present models, the following material properties are adopted under osteoporotic conditions: the elastic moduli of all bone structures are decreased 66% for trabecular bone and 33% for cortical bone.^15^ For cortical bone, the mechanical properties vary as the age increases; the elastic modulus under tension or compression degrades by approximately 2% per decade.^16^ Moreover, the elastic moduli of the cortical bone were found to range from 10 and 20 GPa(Giga Pascals).^17^ Therefore, the elastic modulus may vary between 9.2 and 18.4 GPa with age-related change over 40 years (Table-I).

In the graded models, the cortical bone is divided into two layers with different mechanical properties. In the non-graded models, all of the cortical bone is defined by homogeneous materials with constant mechanical properties. Altogether, four models are constructed in this paper. Model 1 and model 2 belong to Type 1: graded models. Model 3 and model 4 belong to Type 2: non-graded models. Of the graded models, model 1 corresponds to the low elastic modulus group; model 2 corresponds to the high elastic modulus group. In the non-graded models, model 3 corresponds to the low elastic modulus; model 4 corresponds to the high elastic modulus. The material properties for cortical bone are listed in Table-I, and those for the dental implant and trabecular bone are listed in Table-II.

**Table-I:** Young's Modulus (*E*) and Poisson Ratio (*µ*) of the cortical bone used in this study

*Types of models*	*Models*	*E (GPa)*		*µ*
		*Out layer*	*Inner layer*	*Cortical bone*
Type 1 (graded model)^16^^,^^17^	model 1	9.20	6.16	0.30
	model 2	18.40	12.32	0.30
Type 2 (non-graded model)^15^	model 3	6.16	6.16	0.30
	model 4	12.32	12.32	0.30

**Table-II:** Young's Modulus (*E*) and Poisson Ratio (*µ*) of the dental implantand the trabecular bone used in this study.

*Materials*	*E (GPa)*	*µ*
Abutment ^7^	11	0.35
Titanium(Ti) ^7^	11	0.35
Trabecular bone ^15^	0.465	0.30

**Table-III:** Maximum Von Mises stresses and maximum displacements of the cortical bone ingraded models and non-graded models under osteoporotic conditions

Load Direction	*Max Von Mises Stresses * *(MPa)*				*Max* *Displacements (µm)*			
Vertical	4.29~5.14	5.29~6.17	3.16~3.95	4.10~4.92	7.93	5.75	8.73	6.43
Oblique	5.27~6.32	6.55~7.64	4.21~5.26	5.42~6.50	6.67	4.31	7.35	4.82

**Table-IV:** The relative differences of maximum Von Mises stresses and maximum displacements of all the models of the cortical bone under osteoporotic conditions

Load direction	*Max Von Mises stresses*				*Max displacements*			
	*R*_13_	*R*_24_	*R*_12_	*R*_34_	*D*_13_	*D*_24_	*D*_12_	*D*_34_
Vertical	30.1%~35.8%	25.4%~29.0%	20.1%~23.3%	24.6%~29.7%	10.1%	11.8%	37.9%	35.8%
Oblique	20.2%~25.2%	17.5%~20.8%	20.9%~24.3%	23.6%~28.5%	10.2%	11.8%	54.8%	52.5%

**Fig.2:**
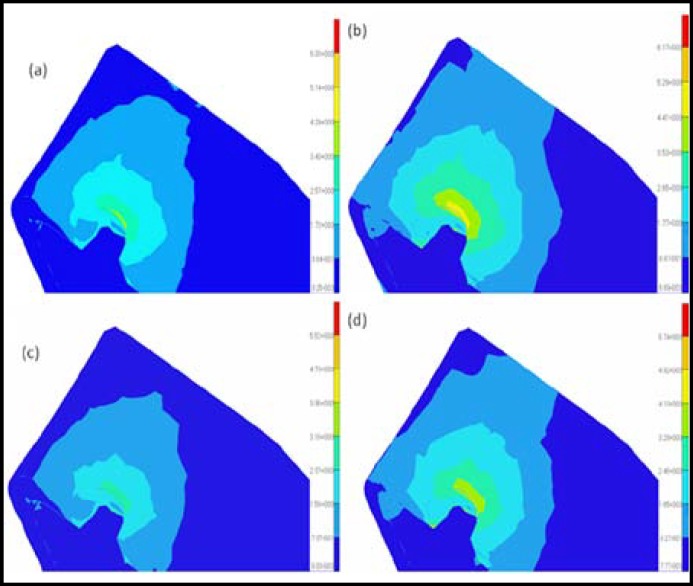
Distributions of the Von Mises stresses in the cortical bone under vertical loading conditions for different models: (a) model 1; (b) model 2; (c) model 3; (d) model 4

**Fig.3:**
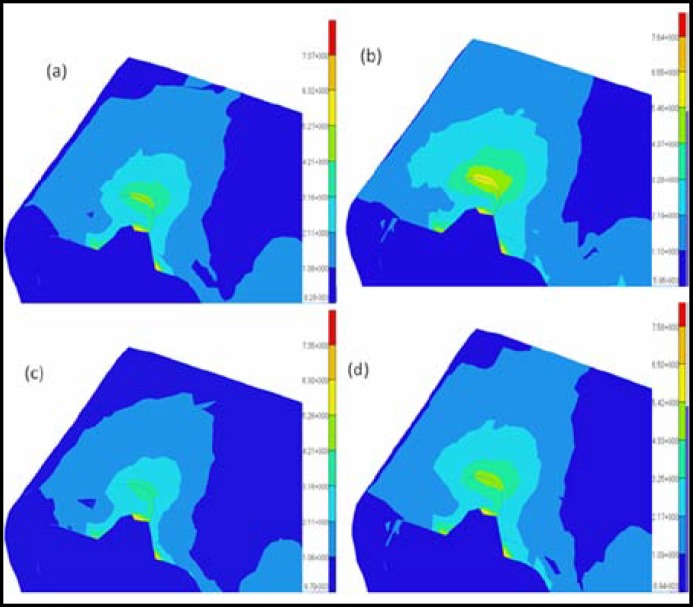
Distributions of the Von Mises stresses in the cortical bone under oblique loading conditions for different models: (a) model 1; (b) model 2; (c) model 3; (d) model 4

**Fig.4:**
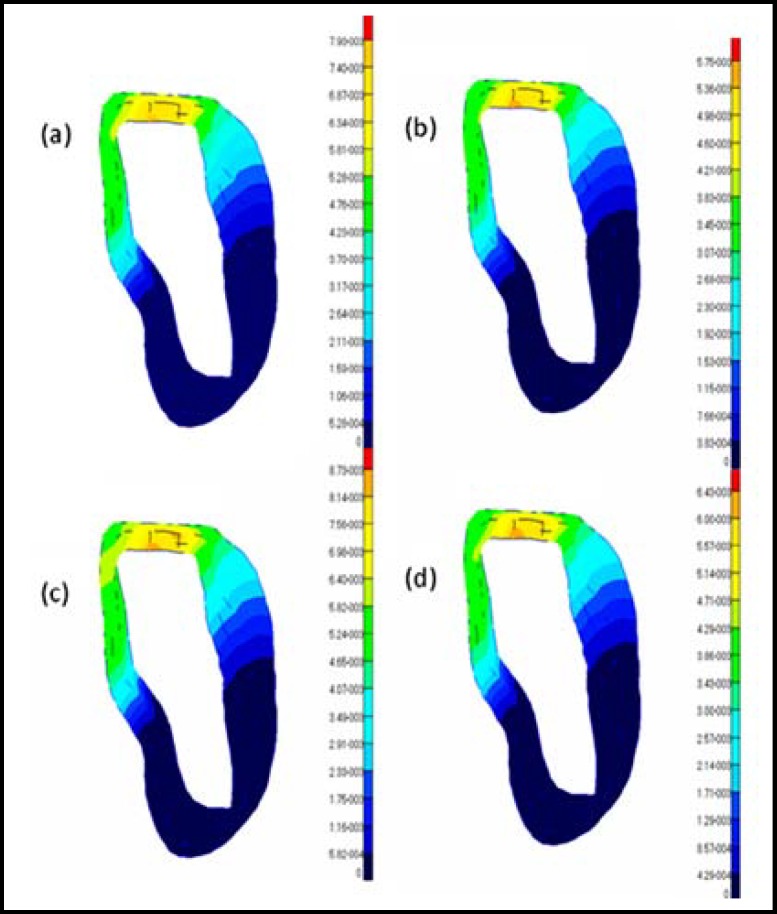
Distributions of the displacements in the cortical bone under vertical loading conditions for different models: (a) model 1; (b) model 2; (c) model 3; (d) model 4

**Fig.5:**
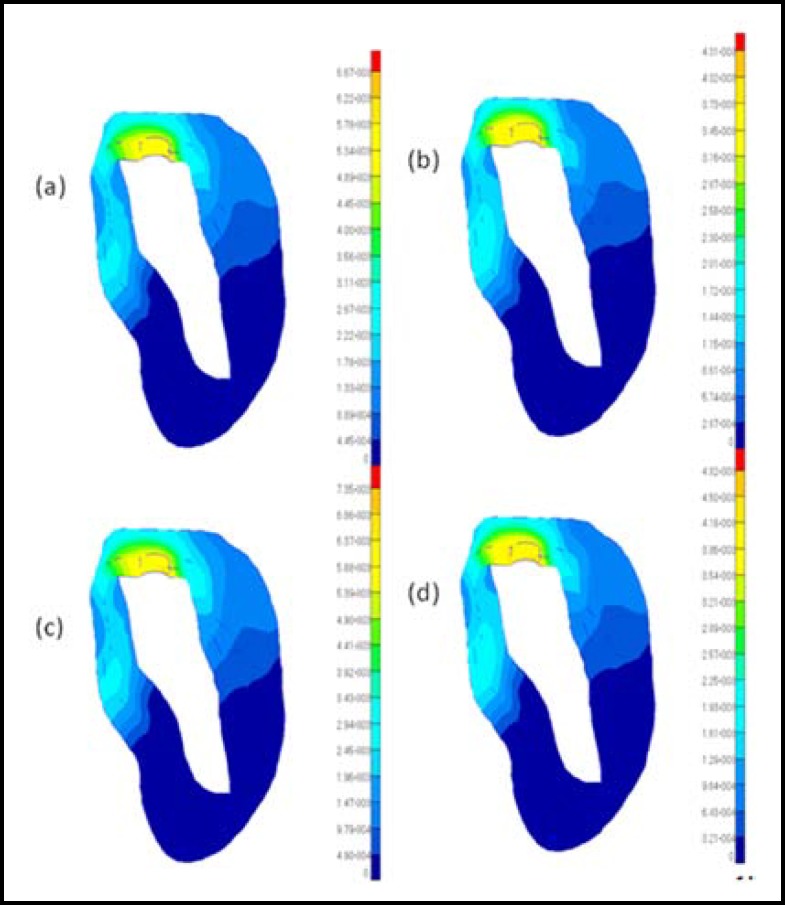
Distributions of the displacements in the cortical bone under oblique loading conditions for different models: (a) model 1; (b) model 2; (c) model 3; (d) model 4

## RESULTS

The results from the graded models of the cortical bone were compared with those from the non-graded models to analyze the effects of the modeling methods of the cortical bone on the bone-implant system under osteoporotic conditions. Because the elastic modulus may decrease with age, the difference in the elastic modulus may reflect the variation of the quality of the cortical bone. Thus, the influences of age on cortical bone can be considered by choosing a different elastic modulus in the graded models or in the non-graded models. The cortical bone stress distributions and maximum displacements of the four models are shown in Fig.2 to 5. The values of the maximum von Mises stresses and the maximum displacements in the cortical bone under vertical and oblique loads are listed in Table-III. The relative differences of the maximum von Mises stresses and the maximum displacements in the cortical bone under vertical and oblique loads are listed in Table-IV.

## DISCUSSION

***R*** ***esults*** ***A*** ***nalysis*** ***: ***It is observed that obvious differences in the maximum von Mises stresses between the graded models and the non-graded models can be found in Fig.2 and 3. In Table-IV, the difference in the maximum von Mises stresses in the crest region of the cortical bone is more than 20% between the graded models and the non-graded models. Specifically, the difference reaches 30% between model 1 and model 3 under vertical loads. Considering the influences of age, the difference of the maximum von Mises stresses in the crest region of the cortical bone is also more than 20% in the graded models or the non-graded models.

Because the deformation of the bone-implant system may affect the service life considerably,^18^ the maximum displacements in two types of models are analyzed. According to the different mechanical properties assumed for the bone, the analysis reveals an expected significant difference in the deformation of the bone–implant system. Fig. 4 and 5 show the comparison of the magnitude of the displacements in the two types of models with different loads. Obviously, the values of the maximum displacements in the graded models are considerably less than in the non-graded models. In Table-IV, the difference of the maximum displacement in the crest region of the cortical bone is approximately 10% between model 1 and model 3 and approximately 11% between model 2 and model 4 under either a vertical load or an oblique load. In particular, the difference in the maximum displacements is more than 50% in the graded models and more than 35% in the non-graded models.

The results of the maximum von Mises stresses and the maximum displacements show obvious differences between the graded models and the non-graded models. These differences may occur because of the different mechanical properties of the cortical bone in the two types of models, because the internal organization structures of cortical bone are continuously changing, cortical bone has graded structures, the graded structures of cortical bone relate to their functions such that they allow the cortical bone to transfer, diminish and cushion the outside force. The problem of the graded property of cortical bone is traditionally solved with averaged mechanical properties.^19^ Despite this solution, some investigators continue to assign homogeneous properties to their models without considering the effect of assumptions on their predictions.

However, the elastic modulus of cortical bone has obvious effects on the stability and long-term success of dental implants. Some studies have indicated that accurate knowledge and the capability of modeling the effective characteristics of bone are essential for reliable investigation of the bone–dental implant system biomechanics.^20^ Therefore, it is important to take into account the graded properties of cortical bone so that the biomechanical behavior of the bone-implant system can be analyzed accurately.

***Crestal*** ***B*** ***one*** ***L*** ***oss and*** ***O*** ***steoporosis: ***Crestal bone loss is the most common factor for the failure of dental implants. In the literature, many biomechanical and biological factors have been identiﬁed as reasons for crestal bone loss.^21^ Essentially, biological factors have been demonstrated in many studies.^22^ However, biomechanically driven crestal bone reactions are still required to qualify and quantify the biomechanical factors leading to crestal bone loss. ^18^ Load-induced crestal bone loss includes occlusal overload and underload conditions. Occlusal overload has been proposed as a potential causative factor that may cause pathological stresses, stimulating crestal bone loss.^23^ In addition, in occlusal underload, lack of stimulus to the bone may cause disused bone loss in the crestal area.

It is well known that the osteoporosis rate increases with age. At the same time, the incidence of missing teeth usually increases dramatically with age. Dental implantation is a widely accepted method for dental restorations. However, osteoporosis of the jawbone often results in the failure of dental implants. Moreover, osteoporosis has been reported to increase crestal bone loss.^18^ Therefore, it is important to accurately simulate the actual loads of the jawbone to analyze the effects of the osteoporosis on the stability and long-term success of dental implants. Therefore, establishing the proper models under osteoporotic conditions is preventively important in understanding the mechanical causes leading to crestal bone loss.

## CONCLUSIONS

This study aimed to investigate the effects of graded models on the biomechanical behavior of the bone-implant system under osteoporotic conditions using the finite element method. Comparing the two types of models, the results of the maximum von Mises stresses and the maximum displacements show obvious differences between the graded models and the non-graded models. This observation indicates the significance of taking into account the actual graded properties of cortical bone so that the biomechanical behavior of the bone-dental implant system can be analyzed accurately.
